# Investigating Multi-Mycotoxin Exposure in Occupational Settings: A Biomonitoring and Airborne Measurement Approach

**DOI:** 10.3390/toxins13010054

**Published:** 2021-01-13

**Authors:** Sophie Ndaw, Daniele Jargot, Guillaume Antoine, Flavien Denis, Sandrine Melin, Alain Robert

**Affiliations:** 1 Toxicology and Biomonitoring Department, INRS—French National Research and Safety Institute for the Prevention of Occupational Accidents and Diseases, 54500 Vandoeuvre-Lés-Nancy, France; guillaume.antoine@inrs.fr (G.A.); flavien.denis@inrs.fr (F.D.); alain.robert@inrs.fr (A.R.); 2 Pollutant Metrology Department, INRS—French National Research and Safety Institute for the Prevention of Occupational Accidents and Diseases, 54500 Vandoeuvre-Lés-Nancy, France; daniele.jargot@inrs.fr (D.J.); sandrine.melin@inrs.fr (S.M.)

**Keywords:** mycotoxins, exposure assessment, biomonitoring, air, dust, HR-MS/MS, occupational exposure

## Abstract

Investigating workplace exposure to mycotoxins is of the utmost importance in supporting the implementation of preventive measures for workers. The aim of this study was to provide tools for measuring mycotoxins in urine and airborne samples. A multi-class mycotoxin method was developed in urine for the determination of aflatoxin B1, aflatoxin M1, ochratoxin A, ochratoxin α, deoxynivalenol, zearalenone, α-zearalenol, β-zearalenol, fumonisin B1, HT2-toxin and T2-toxin. Analysis was based on liquid chromatography–high resolution mass spectrometry. Sample pre-treatments included enzymatic digestion and an online or offline sample clean-up step. The method was validated according to the European Medicines Agency guidance procedures. In order to estimate external exposure, air samples collected with a CIP 10 (Capteur Individuel de Particules 10) personal dust sampler were analyzed for the quantification of up to ten mycotoxins, including aflatoxins, ochratoxin A, deoxynivalenol, zearalenone, fumonisin B1 and HT-2 toxin and T-2 toxin. The method was validated according to standards for workplace exposure to chemical and biological agents EN 482. Both methods, biomonitoring and airborne mycotoxin measurement, showed good analytical performances. They were successfully applied in a small pilot study to assess mycotoxin contamination in workers during cleaning of a grain elevator. We demonstrated that this approach was suitable for investigating occupational exposure to mycotoxins.

## 1. Introduction

Mycotoxins are secondary metabolites of fungi that are known to exert a wide range of toxicities in humans and animals. Depending on the type of mycotoxins, nephrotoxicity, cancer, liver toxicity, impaired immunological functions and growth retardation have been reported as among their adverse health effects [1]. Mycotoxins contaminate many of the most frequently consumed foods and feeds worldwide, including cereals, nuts, dried fruits and spices. The various surveys carried out on the prevalence of mycotoxins in food crops indicate that 60–80% of world production is contaminated [2]. However, in most cases, the levels detected do not exceed regulatory or recommended values that have been in place at national levels [3]. The ingestion of contaminated foodstuffs results in a dietary exposure of the general population. An additional mycotoxin exposure can also occur via inhalation, or through dermal exposure to air and dust that both contain mycotoxins and mainly occur in workplaces.

Occupational exposure may occur in workplaces due to the mycotoxins contained in organic matters, such as feed, food or waste. Studies have reported the prevalence of mycotoxins in airborne dust and settled dust samples in grain industries [4,5,6,7,8], food industries [4,9,10,11,12,13] and farming [14,15]. These findings point to the prevalence of an occupational exposure to mycotoxins through inhalation, skin contact and hand-to-mouth contact.

High exposure to organic dust during the storage, loading, handling or crushing of contaminated materials (grain, food, feed and waste), and other tasks such as the care of farm animals, can be a source of exposure for workers. Mycotoxins in grain dust are mainly contained in the spores of microscopic fungi and fragments of mycelium [16]. Although non-volatile, mycotoxins can be inhaled through these airborne, contaminated particles. Grain dust could present an occupational hazard whereby the protection of workers is inadequate. Studies have shown that the amount of mycotoxins found in dust can be more than ten times greater than that found in raw materials. Indeed, mycotoxins are largely present on the surfaces of raw materials and tend to be adsorbed into the dust during handling [4,7].

While only rarely investigated, the dermal route for mycotoxins cannot be ruled out. Boonen et al. [17] reported that mycotoxins such as aflatoxin B1 (AFB1), ochratoxin A (OTA), zearalenone (ZEN) and T-2 toxin (T-2) can penetrate into and through the skin, suggesting an additional source of exposure in workplaces during skin contact with contaminated particles or substrates.

In this context, the main questions that need to be answered are (1) the extent to which workplace exposure compares to exposure resulting from the ingestion of mycotoxin-contaminated food, and (2) the magnitude of the potential health risk associated with occupational exposure to mycotoxins.

Biomonitoring has proven to be a valuable tool for measuring mycotoxin total body burden at the individual level. Absorption following oral exposure has been extensively explored in the general population [18,19,20,21], while occupational exposure has only scarcely been studied. During these last two decades, studies have mainly focused on occupational exposure to the carcinogenic AFB1 [22,23,24,25,26,27,28,29,30,31,32], OTA [9,10,33,34,35,36] and deoxynivalenol (DON) [37]. Only a small number of studies have reported the use of a multi-biomarker approach to assess exposure to mycotoxins among workers [13,15,38,39,40]. It is, however, known that the co-occurrence of mycotoxins in food and feed is common [41,42,43], and humans are often exposed to more than one mycotoxin at the same time. Furthermore, the combined effects of mixtures of mycotoxins have been reported in several in vitro studies [44,45,46]. It is therefore relevant, from a health perspective, to study the simultaneous exposure to different mycotoxins, especially in occupational settings where exposure scenarios can be highly variable depending on the sectors, materials handled and levels of protective measures in place.

The ability to determine multiple co-occurring mycotoxins in food, feed and biological matrices has expanded significantly since 2010. Progress in liquid chromatography mass spectrometry, combined with an appropriate sample clean-up procedure, has allowed for most mycotoxins to be quantified sensitively [47,48,49].

The aim of this study was to develop air measurement and biomonitoring methods suitable for the assessment of multiple co-occurring mycotoxin exposures in workplaces. The mycotoxins selected, AFB1, OTA, DON, ZEN, fumonisin B1 (FB1), T-2 and HT-2 toxin (HT-2), were considered to be among the mycotoxins of greatest importance. A multi-class mycotoxin method based on liquid chromatography–high-resolution mass spectrometry (LC-HRMS) was developed in urine for the determination of mycotoxin biomarkers AFB1, aflatoxin M1 (AFM1), OTA, ochratoxin α (OTα), DON, ZEN, α- and β-zearalenol (α-and β-ZEL), FB1, HT-2 and T-2. For air measurement, AFB1, aflatoxin B2 (AFB2), aflatoxin G1 (AFG1), aflatoxin G2 (AFG2), OTA, ZEN, FB1, and DON airborne mycotoxins were quantified in dust using a combination of immunoaffinity sample clean-up and liquid chromatography with fluorescence and UV detection methods. Methodological feasibility was demonstrated in a pilot study conducted on workers from a grain elevator.

## 2. Results

### 2.1. Airborne Mycotoxin Determination

#### 2.1.1. Workplace Air Sampling

Several bioaerosol samplers had been tested and compared for their collection efficiency for aeroallergens and aeropathogens [50,51]. It was shown that samplers should collect the inhalable dust fraction, which approximates to the airborne particles that enter the nose and mouth during breathing and can therefore settle in the respiratory tract. The choice and evaluation of the personal aerosol sampler CIP 10, equipped with an inhalable health-related aerosol fraction selector, had been previously discussed and validated within the scope of previously published methods to monitor occupational exposure to OTA, fumonisins, aflatoxins or zearalenone [4]. Using a multipurpose generator system, the CIP 10 sampler was then validated for dust collection from 1 mg to 65 mg [4]. With an allowable dust accumulation up to 65 mg, this sampler was appropriate for low mycotoxin concentrations, as well as having an 8 h sampling time, even in quite dusty atmospheres

#### 2.1.2. Multi-Class Mycotoxin Analysis in Air Samples

With regard to air measurement, our primary goal was to develop a user-friendly method, using conventional analytical equipment such as liquid chromatography coupled to UV/fluorescence detection, to be routinely implemented in workplaces by the partners of the French occupational safety network.

We had previously reported methods for quantifying frequently occurring airborne mycotoxins in the workplace [4]. These methods were successfully validated and met the criteria of reproducible and reliable methods for personal workplace sampling. However, their main drawback was that only one mycotoxin could be determined at one time in each airborne dust sample.

The initial objective in this study was to analyze in one shot several mycotoxins in an airborne sample by performing a single set of clean-up and chromatographic separation using UV and fluorescence detection within the sample.

The use of two immunoaffinity columns (IAC) (AOF MS-PREP^®^ and DZT MS-PREP^®^) connected in tandem has facilitated the selective clean-up for AFB1-2, AFG1-2, OTA, FB1, DON, ZEN, T-2 and HT-2. The initial protocol proposed by Wilcox et al. [52] for food matrices was slightly modified and adapted to the analysis of mycotoxins in airborne dust samples and at a lower sample quantity. The recovery from IAC comprised 104% for T-2 and 24% for FB1. To account for the low recovery of FB1 as well as AFB1-2 and AFG1-2 during sample clean-up, external calibration standard samples, prepared from a mixed standard solution, were subjected to the entire procedure.

An appropriate and reliable chromatographic separation could not be achieved to quantify the selected mycotoxins in a single run, due to the diversity of chemical properties and the high sensitivity needed. DON, T-2 and HT-2 had to be quantified using UV detection, whereas FL detection was more adapted to the quantification of AFB1-2, AFG1-2, FB1, OTA and ZEN. The analysis of FB1 required a pre-column derivatization with o-phthaldialdehyde (OPA)-mercaptoethanol (MCE) reagent. This step was critical because the complex formed between the OPA–MCE reagent and FB1 yielded a fluorescence that began to decrease after 4 min. As for AFB1 and AFG1, a post-column derivatization was needed to increase the sensitivity. A sensitive quantification of the ten selected mycotoxins was achieved by dividing the extract obtained after the sample clean-up step in four aliquots, and performing separate chromatographic analyses on each aliquot.

The analytical recovery rates were tested in accordance with EN ISO 22065 [53] by spiking foam pads with three different quantities of mycotoxin throughout the anticipated air concentration range (maximum, medium, minimum). The mean analytical recovery rates were higher than 75%. The main validation parameters of the airborne mycotoxin analysis method are summarized in Table 1. The limits of quantification (LOQ) for the collecting foam ranged from 30 pg/foam for AFs to 75 ng/foam for T-2/HT-2. For an 8 h sampling duration, the LOQs sat between 0.006 and 15 ng m^−3^. Mycotoxins were stable on the collected foam for up to 30 days at ambient temperature. More validation parameters, including the analytical repeatability, the analytical limits of detection, the linearity range for each analyte and the analytical uncertainty, are accessible in the INRS database for workplace air measurement methods [54,55,56,57,58,59]. The performance of the method met the general requirements set for airborne chemical agent measurement procedures, with an expanded uncertainty of less than 50% for most mycotoxins. The determination of AFs was, however, considered as semi-quantitative because AFs were quantified out of the concentration range of 0.5 to 10 ng mL^−1^ recommended by the IAC supplier. Two more reliable methods for the determination of AFs were developed for the specific quantification of aflatoxins [54,58].

### 2.2. Determination of Urinary Exposure Biomarkers of Mycotoxins

The development of a urinary biomonitoring method included a sample clean-up procedure, LC separation and high-resolution Orbitrap mass spectrometry optimization in order to achieve high sensitivity.

#### 2.2.1. Sample Clean-up

Sample preparation is a crucial step in quantifying mycotoxins in urine samples. It can be challenging because of the diversity of chemical properties possessed by the mycotoxins selected. High sensitivity was important for this application since mycotoxins are expected to be present at low concentrations. Then again, achieving a simple and fast sample clean-up was also important in order to reduce sample analysis time. Three types of sample preparation procedure were investigated to determine which method would result in the cleanest extraction and highest recovery, therefore meeting the limit of quantification requirement. A simple dilute-and-shoot approach was primarily investigated given the analytical performance of high-resolution mass spectrometry. This approach was easy to implement and very convenient. An online sample clean-up and an offline solid phase extraction were also explored. In order to choose an appropriate sample clean-up method with the highest sensitivity, an estimation of the lowest LOQ was performed by analyzing urine samples contaminated with all the mycotoxins and metabolites with concentrations ranging from 5 ng/L to 5 µg/L. The results obtained are shown in Table 2. As Table 2 shows, each sample clean-up method allowed the quantification of all analytes except for DON and HT-2. HT-2 and DON were poorly recovered when using SPE Isolute Myco cartridges and online cyclone P cartridges, respectively. When comparing the three sample clean-up techniques, the lowest LOQs were obtained with the online sample clean-up, except for DON. Due to its high polarity, DON was not adequately retained on the analytical column following elution from the online cyclone P cartridge, giving a broad peak. A better quantification of DON was achieved when using the dilute-and-shoot approach or SPE Isolute Myco extraction, but the LOQ was high, ranging from 1 to 2 µg/L. An improved sensitivity with an LOQ of 0.1 µg/L was obtained for DON with an Oasis HLB SPE cartridge (Table 2). An online sample clean-up procedure was then chosen for all of the targeted metabolites except DON. A separate sample preparation procedure by SPE, using an oasis HLB cartridge, was performed for DON.

#### 2.2.2. Analytical Method Validation

Excellent linearities with a mean R^2^ > 0.990 were obtained for all the calibration curves. The linearity ranges are summarized in Table 3.

The LC-HR/MS method for mycotoxin biomarkers determination combined with an online or offline (for DON) clean-up step was further validated by an in-house validation protocol. Analytical quality control sample results are reported in Table 3. The intra-day accuracy and precision were in the range of 95.4 to 114.5% and 0.6 to 15.1%, respectively. Inter-day accuracy and precision were in the range of 94.0 to 113.1% and 2.7 to 16.4%, respectively. These results comply with the set acceptance criteria of 80–120% accuracy and ≤20% relative standard deviation (RSD).

The LOQs were found to be between 0.01 and 1 µg/L. As can be seen in Table 3, a higher LOQ of 1 µg/L was obtained for α-ZEL and HT-2, while AFB1 and OTA showed the lowest LOQ of 0.01 µg/L.

The online clean-up method provided a high recovery rate for the selected biomarkers, ranging from 88% for ZEN to 116% for FB1 (Table 2). The recovery of DON using Oasis HLB clean-up was 105%. The mycotoxin biomarkers were stable in urine samples for up to 14 days at +4 °C and 6 months at 20 °C.

### 2.3. Pilot Study

Three airborne dust samples were collected from workers. The collection duration was about 6 h. Workers were exposed to very high levels of dust during the cleaning task. Dust concentrations ranged between 29.7 and 105 mg m^−3^ (Table 4). The quantity of dust collected on the foam pad from worker 2 was far above the maximum capacity of 65 mg for which it has been validated. The collecting device was saturated at this level, and the worker exposure was certainly underestimated. Airborne dust samples showed quantifiable levels of DON, AFB1, FB1, OTA and ZEN. AFB2, AFG1-2, T-2 and HT-2 were not detected in any sample. Mycotoxin exposure levels among the workers were highly variable. Concentrations were registered between 28.3 and 108 ng m^−3^ for DON, 80.0 and 120 pg m^−3^ for AFB1, 97.0 and 873 pg m^−3^ for FB1, 38.0 and 194 ng m^−3^ for OTA and 32.1 and 285 ng m^−3^ for ZEN (Table 4).

The presence of DON was detected in 100% of the nine urine samples collected from workers. The median concentration was 14.4 µg/L (Table 5). ZEN and its metabolite αZEL were quantified in 67% and 23% of samples, respectively. OTA was detected in all samples but at lower concentrations compare to DON or ZEN. The concentrations varied from 20.3 to 42.5 ng/L, and its metabolite OTα was not quantified in any urine sample. AFB1 and AFM1 were less frequently quantified than DON, OTA and ZEN, in 55% and 44% of samples, respectively. The concentrations varied from <LOQ to 239 ng/L for AFB1, and the median concentration was 352 ng/L for AFM1. Despite being quantified in airborne dust samples, FB1 was not detected in urine samples. In the same way, βZEL, HT-2 and T-2 were not quantified in any of the nine urine samples collected from workers. All samples contained biomarkers of three to five mycotoxins.

## 3. Discussion

The main objective of this study was to develop sensitive and reliable multi-mycotoxin assays in urine and air dust samples to assess exposure among workers. Common toxicologically important mycotoxins of possible interest in workplaces included AFB1, OTA, ZEN, FB1 and trichothecenes DON, T-2 and HT-2.

The measurement method of airborne mycotoxins included an active air sampling of organic dust particles, together with an immunoaffinity sample treatment, which was part of the well-established protocols for mycotoxin determination in food and feed samples. The conventional liquid chromatography analysis coupled with a UV/FL detection was also convenient for analyzing settled dust or bulk material.

The personal aerosol sampler CIP 10 with its high-efficiency particle size selector, and a fit-for-purpose collection efficiency in the particle size ranges of interest, was fully adapted to monitor occupational exposure to contaminated dust [4,15,60]. This sampler was also convenient for handling, and appropriate for any kind of situation with its 10 L/min airflow rate and the total amount of 65 mg of dust that it could collect. Low mycotoxin airborne concentrations were indeed expected given the regulatory control limits required in the food industry [61,62,63], and the 10 mg m^−3^ occupational airborne limit value for non-specific effect dust. Besides, this sampler was well-suited to measuring exposure over a full work shift, as well as to determining short-term high exposure. It was furthermore adapted to explosive environments, whether in food-processing or grain storage, and is now ATEX-certified in accordance with the Directive 2014/34/EU [64].

The multi-class mycotoxin analysis in air samples was fully validated and a mixture of up to ten mycotoxins in a single air sample could be measured. Compared with our previously published method [4], this constituted a saving in terms of measurement numbers, an analysis time-frame quartered across the board, a reduced volume of solvent and a cost saving of 75% for the IACs, as the previous individual columns had a very limited shelf life, which made their storage and use very difficult.

The limits of quantification in this study could not easily be compared to previously published data due to the discrepancy between method validation parameters when available, and due to the differences between the units used to express data [5,6,12,15,65]. For example, whereas the LOQs in this study were 0.06 ng m^−3^ for OTA, 8 ng m^−3^ for DON, and 1 ng m^−3^ for ZEN in the air, they ranged roughly from 0.01 µg/kg to 20 µg/kg, or were displayed as 0.002 ng in a review published by Viegas et al. [66]. Similar LOQs to those in our study were reported by Niculita-Hirzel et al. [6]. They ranged from 0.12 ng m^−3^ for ZEN to 1.15 ng m^−3^ for DON. Furthermore, information on sampling efficiency was lacking in previously published papers. This highlighted the need for a standardized methodology that should allow for the comparison of data and for research to reach a conclusion concerning the significance of airborne contamination in worker exposure and diseases [66].

Our method was successfully applied to a pilot field measurement campaign to assess the likely occurrence of airborne mycotoxins, and to highlight any possible difficulties linked to mycotoxin air sampling compared to monitoring these in real-life occupational settings.

High method sensitivity is also of utmost importance for mycotoxin determination in urine, since the concentration is often low. Depending on the analytical technique implemented, elaborated extraction procedures from urine may be needed in order to achieve this goal. Another requirement is the capacity to process a large number of samples within a reasonable time for exposure studies. In this study, the sample preparation was kept as simple as possible using a non-time-consuming online extraction approach and a reduced volume of urine for the determination of AFB1, FB1, OTA, ZEN, T-2, HT-2 and their metabolites. For DON, which is a predominant mycotoxin in Europe, a separate sample preparation procedure was performed to achieve a highly sensitive quantification. The high resolution of the LC-Orbitrap HRMS technique used provides a high level of selectivity, and hence a good sensitivity for the quantitative determination of the targeted mycotoxins. This analytical method can also be used in screening the presence of additional mycotoxins and metabolites. The validated method was found to be suitable for measuring low levels of mycotoxins.

A similar analytical technique has been previously described by Slobodchikova et al. [49] for determining 17 mycotoxins and metabolites in human plasma. The authors achieved good sensitivity by using a time-consuming three step liquid extraction procedure. Their method provides better LOQs, ranging from 0.2 to 0.5 µg/L for α-ZEL, β-ZEL, T-2 and HT-2, than in our study, where the LOQs were between 0.5 and 1 µg/L. Then again, better LOQs, up to ten times lower and ranging from 0.01 to 0.05 µg/L, were achieved in our study for DON, ZEN and AFB1, while they reported LOQs between 0.1 and 0.5 µg/L. In addition, Slobodchikova et al.’s method was not suitable for OTA and FB1 due to unacceptable recovery. In addition, precision and accuracy for ZEN and α-ZEL at the LOQ levels did not meet the FDA requirement. The method developed and validated in this study was satisfactory, and gives sensitivity comparable to the multi-class LC-MS/MS methods previously reported for the determination of mycotoxins in urine samples [67,68,69,70], although differences could be observed on the basis of the mycotoxins measured. So far, Sarkanj et al. [48] have reported the lowest LOQs at ng/L levels for the simultaneous determination of DON, AFM1, AFB1, OTA, ZEN, α-ZEL and β-ZEL. However, LOQs were calculated based on a signal to noise ratio of 10:1, rather than the more stringent requirement of ≤ 20% RSD for precision.

The application of the atmospheric measurement and biomonitoring methods in three workers from a grain elevator detected mycotoxins in both airborne and urine samples. Within this pilot study, up to five mycotoxins were determined in air samples, confirming that grain dust could be a source of exposure for workers. Similar observations have been already reported in Europe in grain industries [5,6,7,8]. Quantifiable levels of DON, ZEN and nivalenol have been found in airborne samples collected during wheat harvesting and grain handling in Switzerland [6]. Settled dust collected in grain elevators in Norway [7] and storage facilities in Belgium [8] showed levels of multiple mycotoxins including DON, OTA, ZEN. High incidences of DON, OTA and ZEN have also been reported by Mayer et al. [5] in settled dust in grain elevators in Germany.

The low number of urine samples collected does not enable us to draw any conclusions on the magnitude of the occupational exposure of these workers, or on the efficiency of respiratory protective masks. However, the presence of aflatoxins in half of the urine samples could be an indication of occupational exposure, given that AFB1 is rarely detected in the general population [22]. Overall, these data confirmed the need for multi-mycotoxin methods in assessing mycotoxin exposure among workers. The pilot study demonstrated the applicability of the validated methods and confirmed the relevance of the biomonitoring and airborne measurement approaches for a better understanding of exposure scenarios in occupational settings. This approach opens up new possibilities for large-scale occupational studies to shed light on the contribution of workplaces to mycotoxin exposure in humans.

## 4. Materials and Methods

### 4.1. Airborne Mycotoxin Determination

#### 4.1.1. Chemicals and Reagents

For the preparation of samples, o-phthaldialdehyde (≥97% for HPLC), 2-mercaptoethanol (≥99.0%), potassium bromide (Purum p.a., ≥99.5%) and boron trifluoride BF3 (10–20% in methanol) were from Sigma-Aldrich (Saint-Quentin Fallavier, France). Purified water (18 MΩ cm) was produced in-house from an Academic MilliQ model water purification unit (Millipore, EMD Millipore Corporation, Billerica, MA, USA). For the preparation of LC mobile phases, acetonitrile (RS for isocratic HPLC, Carlo-Erba Reagents, Val-de-Reuil, France) and methanol (Lichrosolv for HPLC, Merck, Darmstadt, Germany) were used. All other solvents and reagents were of analytical grade.

The mycotoxin primary standard solution of OTA (10 µg/mL in acetonitrile) was from Sigma-Aldrich Chemie (Schnelldorf, Germany). FB1 and FB2 together (50 µg/mL each in an acetonitrile–water mixture) were from Romer labs (Getzersdorf, Austria). Aflatoxins B1, B2, G1 and G2 (250 ng/mL of each in acetonitrile) were purchased from Libios (Bully, France) and T-2 Toxin (100 µg/mL in acetonitrile), HT-2 Toxin (100 µg/mL in acetonitrile), DON (100 µg/mL in methanol) and ZEA (25 µg/mL in methanol) were procured from R-Biopharm (Saint-Didier-au-Mont-d’Or, France).

#### 4.1.2. Air Sampling

Full shift airborne dust samples were collected at the workplace with a CIP 10 personal dust sampler (Tecora, Fontenay Sous Bois, France) designed and patented by INRS. The CIP 10 was equipped with a rotating filter cup containing a polyurethane collecting foam and a particle size selector for the inhalable aerosol fraction (CIP 10-I sampling unit). The airflow was 10 L min^−1^. Before use, each sampler was calibrated on a test rig using pressure drop compensation and its stability was estimated by checking the cup rotation speed with an ARC 8527 tachometer (Tecora, Fontenay Sous Bois, France).

To identify any background contamination and to validate the air sampling step, field blank samples, consisting of foam pads in their cup, were also analyzed.

#### 4.1.3. Sample Preparation

An AX26 balance (Mettler Toledo, Viroflay, France), accurate to the microgram scale, was used for the gravimetric determination of the dust in the disassembled rotating cup in controlled temperature and hygrometry conditions.

After weighing, the dust collected on the foam filter pad was recovered with 10 mL of a water/methanol mixture (30/70). The dust deposited on the inner surfaces of the cup was further collected with 3 mL of the same solvent mixture. The whole sample extract was then diluted with 160 mL of phosphate buffer saline solution (PBS), filtered through filter paper and applied to immunoaffinity columns AOF MS-PREP^®^ and DZT MS-PREP^®^ (R-Biopharm, Saint-Didier-au-Mont-d’Or France) connected in tandem at a steady flow rate of 2 mL per minute under gravity, or by applying a maximum 5-bar vacuum. The columns were washed with 20 mL of water and any residual liquid was removed before elution. The analytes were eluted under gravity at a flow rate of 1 drop per second, with two portions of 1 mL of 100% methanol. The eluate was collected in a tared vial to give a 2 mL total volume, verified by the vial weighing, and subsequently concentrated to dryness under a gentle stream of nitrogen. The dry residue was dissolved in 500 µL of a water/methanol mixture (50/50) and divided into four identical 120 µL aliquots for mycotoxin quantification. The volume of each aliquot was further gravimetrically determined using an analytical XP205DR balance (Mettler Toledo, Virofaly, France).

#### 4.1.4. Analytical Procedure

An overview of the analytical workflow is provided in Figure S1. Separate analyses were performed on each aliquot on a liquid chromatography (LC) system (Perkin Elmer, Series 200) equipped with a binary pump, a vacuum degasser, an auto-sampler, a column oven, a fluorescence detector (FL detector) and a UV detector (Shimadzu). Fumonisin separation was accomplished on an Alltima-HP Reversed-Phase C18 column (5 µm, 150 mm, 3 mm I.D., Alltech) at a temperature of 40 °C. AFs, DON, OTA, ZEN, T-2 and HT-2 separation was performed on a GraceSmart-RP18 column (5 µm, 250 mm, 4.6 mm I.D., Grace Davidson Discovery Sciences) at a temperature of 20 °C.

The first aliquot was analyzed for DON, HT2 and T2 in two subsequent injections on the GraceSmart column. The LC parameters for DON were an 80/20 water/methanol mixture as the mobile phase; flow rate 1 mL/min; 20 µL injection volume; the UV detector was set to a wavelength of 220 nm. The LC parameters for T2 and HT2 were a 40/60 water/methanol mixture as the mobile phase; flow rate 1 mL/min; 80 µL injection volume; the UV detector was set to a wavelength of 202 nm.

The second aliquot was analyzed by fluorescence detection for OTA and aflatoxins after a post-column derivatization achieved on the electrochemical cell (KobraCell^®^, R-Biopharm, Saint-Didier-au-Mont-d’Or, France) to enhance the fluorescence of AFB1 and AFG1. The FL detector was set to an excitation wavelength of 330 nm and an emission wavelength of 460 nm. The mobile phase A was a 60/20/20 water/methanol/acetonitrile mixture, with potassium bromide (119 mg/L) and nitric acid (350 μL/L), and mobile phase B was a 40/10/50 water/methanol/acetonitrile mixture with potassium bromide (119 mg/L) and nitric acid (350 μL/L). The gradient started at 1 mL/min with 100% A for 16 min then the percentage of B was increased from 0 to 100% in 1 min, and maintained for 12 min. The mobile phase was then adjusted to its initial conditions to allow us to re-equilibrate the analytical column for 5 min. The total run time was 34 min. The sample volume injected was 80 µL.

ZEN was analyzed in the third aliquot by fluorescence detection with the FL detector operating at an excitation wavelength of 274 nm and an emission wavelength of 446 nm. The chromatographic separation was achieved using the GraceSmart column. The mobile phase was a mixture of 75/25 methanol/water. The flow rate was set to 1 mL/min and the sample volume injected was 80 µL.

For the analysis of FB1 in the last aliquot, a pre-column derivatization was performed in the auto-sampler with an o-phthaldialdehyde/2-mercaptoethanol (OPA-MCE) reagent. The OPA–MCE reagent was prepared by mixing 1 mL of a 40 mg/mL OPA solution in methanol with 50 µL of MCE and 5 mL of a 0.1 M sodium borate decahydrate in water and this was used within 24 h. For the derivatization, 10 µL of OPA–MCE reagent were added to the 120 µL of sample aliquot and mixed, then 80 µL was injected into the LC system within 3 min. Chromatographic separation was performed on the Alltima-HP column heated to 40 °C. The mobile phase consisted of a mixture of 75/25 methanol/0.1 M sodium dihydrogen phosphate solution adjusted to pH 3.35 with phosphoric acid (85%). The flow rate was 0.5 mL/min and the FL detector operated at an excitation wavelength of 335 nm and an emission wavelength of 440 nm.

Quantification was performed by external calibration using a mixed standard solution with a final concentration of 0.625 ng/mL AFs, 0.99 ng/mL OTA, 250 ng/mL ZEN, 800 ng/mL DON, 750 ng/mL HT-2, 1500 ng/mL T-2 and 600 ng/mL FB1. Five standard calibration solutions covering appropriate ranges of analyte concentrations were prepared by further diluting the mixed standard solution in PBS. The standard calibration solutions were also submitted to the entire procedure to overcome mycotoxin recoveries, which can be variable depending on the immunoaffinity column batch. The ranges of mycotoxin concentrations in the calibration aliquot were 60–600 pg/mL for AFs, 100–1000 pg/mL for OTA, 25–250 ng/mL for ZEN, 80–800 ng/mL for DON, 75–750 ng/mL for HT-2, 150–1500 ng/mL for T-2 and 60–600 ng/mL for FB1. Laboratory blank samples were also analyzed for any background contamination.

For data analysis and processing, Perkin Elmer Chromera software was used. The mycotoxin concentrations were adjusted for any positive laboratory blank samples.

#### 4.1.5. Validation Parameters

The method was validated according to in-house specifications for the development of exposure assessment methods and in accordance with EN 482 requirements, over the widest possible but most technically and reasonably practicable dust and mycotoxins range [60]. The validation parameters included sampling efficiency, air sample storage and analytical recovery.

For the recovery studies, certified flour, Trilogy^®^ multi-toxin reference material and BIPEA DON reference material were used. The foam pads were spiked with known quantities of contaminated flour according to the procedure previously described by Jargot et al. [4]. IAC sample clean-up recoveries were estimated in six replicated samples by calculating the concentration ratio between spiked samples and calibration solutions. The efficiency of dust collection from the foam pads was determined by analyzing the reference material spiking solution and comparing the concentrations calculated from the spiked foam pad samples. The limits of quantification (LOQs) were estimated as a signal to noise ratio of 10, then further validated with at least six replicates, as the lowest concentration quantified with an RSD ≤20%. To perform the stability study, foam pads were spiked with reference material at three different concentrations of mycotoxin and stored at room temperature for thirty days.

### 4.2. Determination of Urinary Biomarkers of Mycotoxins

#### 4.2.1. Chemicals and Reagents

Mycotoxin standards of Aflatoxins B1 (AFB1) and Aflatoxin M1 (AFM1) were purchased from Merck KGa (Darmstadt, Germany). Mycotoxins, metabolites and internal standards of HT2 Toxin, T2 Toxin, Ochratoxin A (OTA), Deoxynivalenol (DON), Fumonisin B1 (FB1), Zearalenone (ZEN), Ochratoxin alpha (OTα), alpha and beta zearalenol (αZEL and βZEL), ^13^C15 Deoxynivalenol (^13^C15-DON), ^13^C17 Aflatoxin B1 (^13^C17-AFB1), ^13^C20 Ochratoxin A (^13^C20-OTA) and ^13^C34 Fumonisin B1 (^13^C34-FB1) were purchased from Biopure™ Romer Labs Division Holding GmbH (Getzersdorf, Austria).

For the preparation of LC mobile phases, mass spectrometry grade acetonitrile and formic acid were obtained from Fisher (Illkirch, France). Ultra-pure water was produced in-house by a Milli-Q^®^ advantage system (Merck KGaA, Darmstadt, Germany). β-glucuronidase from *E. coli*-type IX-A and ammonium Acetate ACS reagent were purchased from Merck KGa. All other solvents and reagents were of analytical grade.

A 1 mg/L stock standard solution, including all the mycotoxins and metabolites with the exception of DON, was made by the adequate dilution of commercial standards in acetonitrile. A 1 mg/L stock solution of internal standards was also prepared in acetonitrile. Stock solutions were stored at −20 °C.

A 1 mg/L stock solution of DON was prepared from commercial solution in water and stored at +4 °C. This stock solution was freshly prepared every 4 months.

Working solutions of mycotoxin standards were daily prepared by diluting the stock solution with pure water. Appropriate serial dilutions of intermediate solutions with a pool of blank human urine were prepared just before the use for calibration in the range 0.01–20 μg/L.

Working solutions of ^13^C-isotopically labeled internal standards (^13^C20-OTA, ^13^C34-FB1 and ^13^C17-AFB1, 25 µg/L) were prepared in water with 0.1% formic acid by diluting stock solution.

A working solution of β-glucuronidase was prepared each day. A volume of 600 μL of a 50,000 U/mL enzyme solution was mixed with 3.4 mL of water and 6 mL of 1 M sodium acetate buffer (pH 5).

#### 4.2.2. Sample Preparation

Urine samples were hydrolyzed with β-glucuronidase enzyme to hydrolyze the glucuronide and/or sulphate conjugates of mycotoxins. For the deconjugation of DON, 600 μL of the working solution of β-glucuronidase, 80 μL of internal standard (^13^C15 DON, 1 mg/L) and 60 µL of water were added to 3 mL of urine sample in a glass vial. For the deconjugation of the other mycotoxins, 200 μL of the working solution of β-glucuronidase, 40 μL of the working solution of internal standards and 60 µL of water were added to 1 mL of urine sample in a glass vial. Urine samples were vortexed, incubated overnight at 37 °C and then cooled down to room temperature.

##### Offline Sample Clean-up

Hydrolyzed urine sample (2 mL) was loaded onto a solid phase extraction cartridge (Oasis^®^ HLB 3 cc 60 mg, Waters, Milford, USA; Isolute^®^myco 3 cc Biotage, Uppsala, Sweden) previously conditioned with 2 mL of methanol and 2 mL of water. The washing steps were 2 mL of ultrapure water and 2 mL of methanol/water (5/95), successively. The analytes were eluted with 3 mL of methanol/water (40/60). The purified extract was evaporated to dryness under a stream of nitrogen. The residue was re-dissolved in 1 mL of ultrapure water and a volume of 20 µL was analyzed by LC-HRMS/MS.

##### Online Sample Clean-up

An online Turboflow™ sample clean-up was developed on a Thermo Scientific Transcend TLX-Q-Exactive system. Two liquid chromatography pumps were combined, with a fluidic set and valves, to build the system. One pump was dedicated to the online SPE Turboflow™ 0.5 × 50 mm Cyclone-P cartridge (Thermo Scientific) and the other was dedicated to the analytical column. Mobile phases for the on-line SPE system were 0.1% formic acid in water (A), 0.1% formic acid in acetonitrile (B) and acetonitrile/methanol/acetone (45/45/10) (D). Hydrolyzed urine sample (100 µL) was run on the Cyclone-P column at a flow rate of 1.5 mL/min of phase A for 1 min. The loop content, composed of 100 µL of 70/30 A/B, was used in a backflush mode to elute and transfer analytes into the analytical column. The Cyclone-P column was then successively washed with 100% of phase B and 100% of phase D.

##### Dilute-and-Shoot Sample Preparation

For the dilute-and-shoot method, the hydrolyzed urine sample was diluted 10 times in ultrapure water and a volume of 10 µL was analyzed by HR-MS/MS.

#### 4.2.3. HR-MS/MS Analysis

The samples were analyzed on a UHPLC–HR-MS/MS system. Chromatographic separation was achieved using a Dionex Ultimate 3000 (Thermo Scientific) liquid chromatography system equipped with a degasser, an autosampler, LC pumps and a column oven. Mycotoxin separation was performed with an Accucore RP-MS column (150 × 2.1 mm, 2.6 µm, Thermo Scientific) at a temperature of 23 °C. The mobile phases consisted of a gradient of 0.1% formic acid in water (A) and 0.1% formic acid in acetonitrile (B), at a flow rate of 0.4 mL/min.

##### Multi-Mycotoxin Method

The linear gradient started at 0.4 mL/min with 95% A and 5% B for 60 s, then the flow was decreased to 0.2 mL/min at 15 s to allow the transfer of analytes from the Turboflow column, for 30 s. Then a composition of 95/5 A/B at 0.4 mL/min was maintained for 90 s before the start of a linear ramp gradient up to 70% of phase B within 6 min. The percentage of 70% B was maintained for 2 min. The mobile phase was then adjusted to its initial conditions to allow us to re-equilibrate the analytical column for 105 s. The total run time was 13 min. The sample volume injected was 100 µL.

The UHPLC system was coupled to a Q-Exactive™ benchtop mass spectrometer (Thermo Scientific, San Francisco, CA, USA) equipped with a heated electrospray ionization source (HESI II) operating in positive mode. The optimized ionization source parameters were as follows: a spray voltage of 3.5 kV; sheath gas at 45 a.u. (arbitrary units); auxiliary gas at 20 a.u.; sweep gas at 0 a.u.; capillary temperature of 250 °C; and auxiliary gas temperature of 350 °C. The optimal MS parameters were S-Lens Radio Frequency (RF) level 50 operating with multiplexed events of targeted single ion monitoring (tSIM) and data-dependent fragmentations (ddMS2). Due to co-eluting mycotoxins and internal standards, a multiplexed tSIM-ddMS2 method with an inclusion list was applied to obtain the highest number of scans for each chromatographic peak and to gain a better sensitivity. Detection was performed using a scan range, multiplexing count and normalized collision energy (NCE) adapted for each event and depending on the analyte retention times. Event 1 (6.5 to 7.5 min) included AFM1, FB1 and ^13^C34-FB1, a scan range of 300 to 770 m/z, a multiplexing count of 3 and an NCE of 35%. Event 2 (7.5 to 9 min) included OTα, AFB1, α-ZEL, β-ZEL, ^13^C17-AFB1 and HT-2, a scan range of 250 to 340 m/z, a multiplexing count of 5 and NCEs of 20% and 35%. Event 3 (9 to 10 min) included ZEN, OTA, ^13^C20-OTA and T-2, a scan range of 300 to 500 m/z, a multiplexing count of 4 and an NCE of 35%. Other detection parameters were common to three events. For the SIM acquisitions, the parameters were as follows: resolution of 70,000 FWHM (full width at half maximum); AGC (automated gain control) target of 5 × 10^4^; maximum injection time (IT) of 75 ms; loop count of 5; quadrupole isolation width of 1 m/z; and a profile type for spectrum data. For the ddMS2 acquisitions, the parameters were as follows: resolution of 35,000 FWHM; AGC target of 5 × 10^4^, IT of 200 ms; loop count of 1; MSX count of 1; top N 1; quadrupole isolation width of 1 m/z; profile type for spectrum data. An intensity threshold of 2.5 × 10^3^ and a dynamic exclusion of 1 s were applied to trigger the MS2 spectrum acquisition.

##### DON Method

The gradient started at 0.4 mL/min with 95% A and 5% B for 2 min. The percentage of B was increased to 37.5% over 3 min then instantly increased to 70% and maintained at this percentage for 30 s. The mobile phase was then adjusted to its initial conditions to allow us to re-equilibrate the analytical column for 2 min. The total run time was 7.5 min. The sample volume injected was 20 µL. The optimized ionization source parameters were as follows: spray voltage of 3.5 kV; sheath gas at 45 a.u.; auxiliary gas at 40 a.u.; sweep gas at 1 a.u.; capillary temperature of 250 °C; and an auxiliary gas temp of 350 °C. The optimal MS parameters were an S-Lens RF level of 50 operating with multiplexed events of targeted single ion monitoring (tSIM) and data-dependent fragmentations (ddMS2). Detection was performed for tSIM acquisition using a scan range of 150 to 400 m/z, resolution of 70,000 FWHM, AGC target 5 × 10^4^, IT 250 ms, loop count 1, MSX count 2, quadrupole isolation width 1 m/z and profile type of spectrum data. For ddMS2 acquisition, the resolution was 35,000 FWHM, AGC target 5 × 10^4^, max IT 200 ms, loop count 2 and MSX count 1, top N 2, NCE 35%, isolation width 1 m/z and a profile type of spectrum data. An intensity threshold of 1.3 × 10^4^ and dynamic exclusion of 1 s were applied to trigger MS2 spectrum acquisition.

The exact mass of the compounds studied, the retention times and the confirmation fragments for the detected ions are provided in Table S1. A mass tolerance of 5 ppm was set for identification and confirmation. For data analysis and processing, the TraceFinder software 4.0 was used. Mycotoxins were quantitated using the precursor ions, which were extracted with a ±5 ppm window.

#### 4.2.4. Validation Parameters

The method of quantification of targeted urinary mycotoxins using LC-Q-orbitrap-MS analysis was evaluated by an in-house validation protocol that was adapted from the procedures described in the FDA guidance for bioanalytical method validation [71]. The main parameters evaluated included linearity, accuracy, precision, stability, recovery and LOQ. Acceptance criteria of 80–120% accuracy and ≤20% RSD were applied. For the linearity, matrix-matched calibration solutions were analyzed by spiking blank urine samples at thirteen concentration levels from 10 ng/L to 50 µg/L. Linearity was evaluated by calculating the mean squared correlation coefficient (R^2^) of three matrix-matched calibration curves for each analyte. For the determination of the method’s intra-day/inter-day accuracy and precision, quality control (QC) samples spiked at three different concentrations were analyzed. Intra-day precision (RSD) was expressed as the relative standard deviation after three determinations in triplicate in a single day. Inter-day precision was calculated by repeating the measurements in triplicate on three non-consecutive days and expressed as relative standard deviation. For the sample clean-up recovery studies, area ratios between purified and non-purified spiked urine samples were calculated. The definition of limit of detection and quantification is not applicable for high-resolution mass chromatography because of the limited noise. Therefore, the LOQ in this method was the lowest concentration quantified with an RSD ≤20%. LOQ was determined based on the results obtained during intra- and inter-day experiments. To perform the stability study, urine samples were spiked with mycotoxins at five different concentration levels from 0.25 to 20 µg/L, and aliquots were stored at four different temperatures (ambient, +10 °C, +4 °C and +20 °C) for further analyses for up to 6 months.

### 4.3. Pilot Study

The pilot study was conducted in a grain elevator on a population of grain workers exposed to dust generated when handling grain. Workers were informed about the investigation and gave their written consent before inclusion in the study. The internal ethical committee approved the study. Grain workers were asked to provide urine samples and to wear personal air sampling devices during their shift. Three male workers were investigated. They were involved in the cleaning of the empty grain dryers for maize and barley. Their main tasks consisted of removing any grain or debris that had accumulated and attached to the sides and floor of the dryers. FFP2 respiratory protection masks were worn throughout the shift. Full shift personal airborne dust samples were collected from the workers’ breathing zones. Dust samples were collected on pre-weighted foam pads using the CIP 10 personal aerosol sampler with its inhalable health-related aerosol fraction selector. Foam pads were stored at room temperature for up to one month until analysis. Spot urine samples, including pre-shift and post-shift samples and first morning void on the following day, were collected from each participant. The urine samples were stored after collection at −20 °C until analysis.

Urinary creatinine was measured in urine samples using the Jaffé colorimetric method.

## Figures and Tables

**Table 1:** Validation parameters of the airborne mycotoxin analysis method.

Mycotoxins	Analytical Recovery (%)	LOQ *** in Solution	LOQ on the Collecting Foam	LOQ in the Air for 8-h Sampling
OTA	97	130 pg mL^−1^	70 pg	0.014 ng m^−3^
FB1	99	50 ng mL^−1^	30 ng	6 ng m^−3^
DON	87	50 ng mL^−1^	30 ng	6 ng m^−3^
ZEN	89	10 ng mL^−1^	5 ng	1 ng m^−3^
T-2	102	140 ng mL^−1^	75 ng	15 ng m^−3^
HT-2	86	140 ng mL^−1^	75 ng	15 ng m^−3^
AFB1 *	76	60 pg mL^−1^	30 pg	0.006 ng m^−3^
AFB2 *	92	60 pg mL^−1^	30 pg	0.006 ng m^−3^
AFG1 *	ND **	60 pg mL^−1^	30 pg	0.006 ng m^−3^
AFG2 *	ND	60 pg mL^−1^	30 pg	0.006 ng m^−3^

* Semi-quantitative analysis between 30 pg and 300 pg/foam. ** ND: Not determined. *** LOQ: limit of quantification.

**Table 2:** Estimated LOQ values and method recoveries for mycotoxin biomarkers in human urine samples obtained with different sample clean-up procedures.

				Sample Clean-Up Procedure				
	Dilute-and-Shoot		SPE Isolute^®^ Myco		SPE HLB		On-Line Cyclone^®^ P	
	LOQ (µg/L)	Recovery (%)	LOQ (µg/L)	Recovery (%)	LOQ (µg/L)	Recovery (%)	LOQ (µg/L)	Recovery (%)
AFB1	0.5	-	0.1	72	-	-	0.01	111
AFM1	0.5	-	0.5	76	-	-	0.05	116
ZEN	2	-	0.5	83	-	-	0.025	88
αZEL	2	-	0.5	68	-	-	1	104
βZEL	2	-	1	81	-	-	0.5	97
OTA	0.1	-	0.5	82	-	-	0.01	88
OTα	0.1	-	0.5	118	-	-	0.25	111
FB1	0.5	-	0.5	62	-	-	0.25	116
DON	2	-	1	87	0.1	105	ND *	-
T-2	5	-	0.1	96	-	-	0.5	105
HT-2	10	-	ND *	-	-	-	1	109

* ND: Not detected.

**Table 3:** Validation parameters of LC-HRMS method using the on-line clean-up procedure.

Mycotoxins	Spike Level µg/L	Intra-Day		Inter-Day		Linear Range µg/L (R^2^)	LOQ µg/L
		Accuracy (%)	RSD %	Accuracy (%)	RSD %		
AFB1	0.05	100.7	2.6	108.3	8.3	0.01–20	0.01
	0.25	104.4	2.9	103.9	8.3	(0.994)	
	2	105.3	1.4	103.9	5.7		
AFM1	0.25	105.4	5.5	99.3	6.1	0.05–20	0.05
	1	98.3	3.0	103.8	3.7	(0.994)	
	5	100.5	3.3	108.7	9.0		
DON *	0.2	107.7	8.5	112.4	16.4	0.05–50	0.05
	1	100.8	4.1	94.5	9.4	(0.999)	
	5	95.4	4.1	94.0	3.9		
FB1	0.25	110.3	5.1	98.8	10.9	0.25–20	0.25
	1	96.9	0.9	99.2	7.4	(0.995)	
	5	99.0	2.0	108.7	8.5		
OTA	0.25	106.5	2.7	104.4	13.6	0.01–20	0.01
	1	106.5	1.7	104.6	4.2	(0.998)	
	5	108.7	1.7	113.1	8.7		
OTα	0.25	107.5	8.9	97.2	10.8	0.25–20	0.25
	1	11.6	3.4	106.8	3.9	(0.996)	
	5	102.7	2.7	106.1	9.7		
ZEN	0.25	101.7	1.6	106.2	9.9	0.025–20	0.025
	1	102.0	0.7	100.8	4.3	(0.998)	
	5	105.5	1.9	111.3	13.9		
αZEL	1	97.8	5.3	105.4	12.8	1–20	1
	5	97.6	2.1	109.9	6.9	(0.990)	
	20	105.6	1.7	102.3	2.7		
βZEL	1	97.0	1.2	98.3	3.8	1–20	0.5
	5	101.2	1.3	104.7	9.0	(0.995)	
	20	102.4	0.6	101.1	4.1		
T-2	0.5	116.0	15.1	106.1	11.9	0.5–20	0.5
	1	111.3	9.1	102.5	7.4	(0.997)	
	5	114.5	1.6	105.5	6.5		
HT-2	2	99.6	5.2	103.5	9.6	1–20	1
	5	105.1	3.0	102.0	7.6	(0.996)	
	20	105.6	2.8	100.8	3.5		

* Offline solid phase extraction using oasis HLB cartridge.

**Table 4:** Dust and mycotoxin concentrations in airborne samples.

	Worker 1	Worker 2	Worker 3
Sampling duration (min)	375	390	380
Airborne dust (mg m^−3^)	48.2	105	29.7
DON (ng m^−3^)	59.1	108	28.3
AFB1 (pg m^−3^)	80.0	120	80.0
FB1 (pg m^−3^)	225	873	97.0
OTA (ng m^−3^)	38.0	194	<LOD
ZEN (ng m^−3^)	155	285	32.1

**Table 5:** Results of mycotoxin biomarkers in worker urine samples (*n* = 9).

	DON	AFB1	AFM1	OTA	ZEN	α-ZEL
**Incidence**	9/9	5/9	4/9	9/9	6/9	2/9
**Uncorrected for creatinine**	µg/L	ng/L	ng/L	ng/L	ng/L	µg/L
**Median**	14.4	20.1	352	24.6	155	-
**Mean**	12.0	98.9	316	27.7	176	1.19
**Minimum**	3.9	<LOQ	<LOQ	20.3	<LOQ	<LOQ
**Maximum**	18.8	239	460	42.5	364	1.20
**Corrected for creatinine**	µg/g	ng/g	ng/g	ng/g	ng/g	µg/g
**Median**	12.1	16.9	300	19.3	119	-
**Mean**	10.8	57.6	413	26.4	107	0.63
**Minimum**	2.75	<LOQ	<LOQ	10.2	<LOQ	<LOQ
**Maximum**	21.4	143	979	76.0	167	0.77

## Data Availability

The data presented in this study are available on request from the corresponding author. The data are not publicly available due to company privacy.

## Supplementary Materials

The following are available online at https://www.mdpi.com/2072-6651/13/1/54/s1, Figure S1: Overview of the sample preparation and sample analysis method for the determination of mycotoxins in airborne samples, Table S1: The monoisotopic masses of the most intense positive ions, retention times and confirmation fragments of mycotoxins and internal standards.
