# Supplementary material for: Investigating Multi-Mycotoxin Exposure in Occupational Settings: A Biomonitoring and Airborne Measurement Approach
Source: Toxins (Basel). 2021 Jan 13;13(1):54. doi: 10.3390/toxins13010054 (PMC7828332; doi:10.3390/toxins13010054)
Supplement: Supplementary file 1 [file toxins-13-00054-s001.pdf]

Supplementary Materials: Investigating Multi-Mycotoxin Exposure in Occupational Settings: A Biomonitoring and Airborne Measurement Approach

Sophie Ndaw, Daniele Jargot, Guillaume Antoine, Flavien Denis, Sandrine Melin and Alain Robert

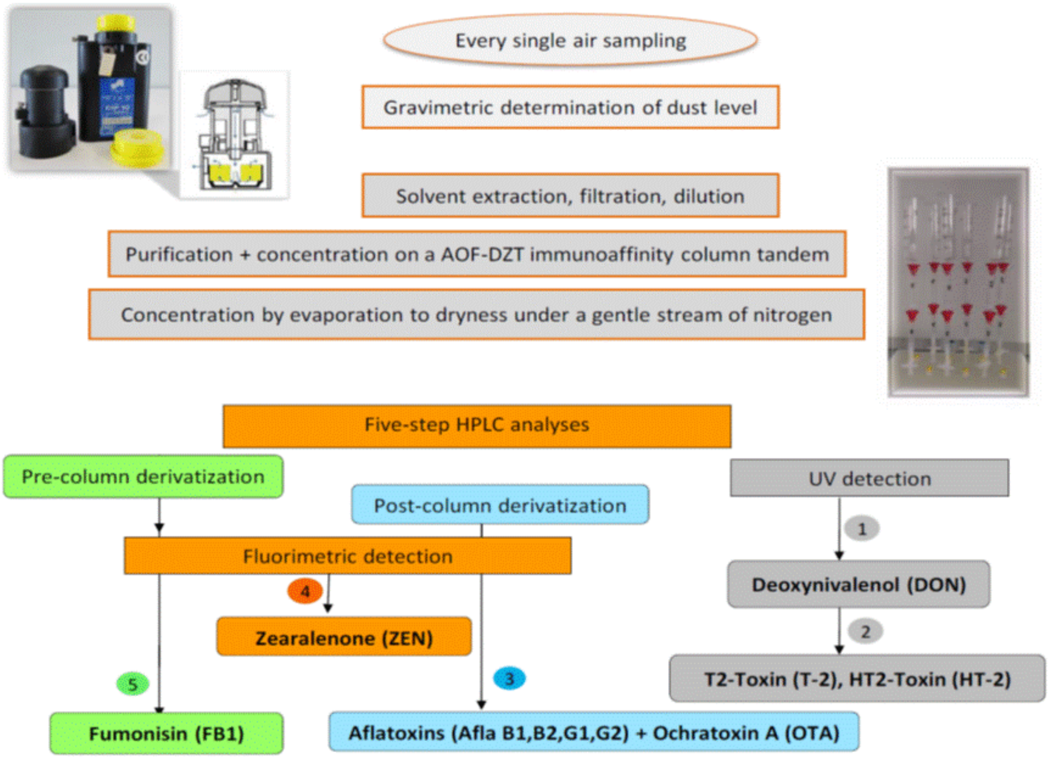

Figure S1. Overview of the sample preparation and sample analysis method for the determination of mycotoxins in airborne samples.

Table S1. The monoisotopic masses of the most intense ions in positive mode, retention times and confirmation fragments of mycotoxins and internal standards.

	Elemental composition	RT, min	The most intense ion	Theoretical m/z	Confirmation fragments
**AFM1**	$C_{17}H_{12}O_7$	7.13	$[M+H]^+$	329.06558	273.0756
**FB1**	$C_{34}H_{59}NO_{15}$	7.3	$[M+H]^+$	722.39575	334.3105
**U-13C34-FB1**	$^{13}C_{34}H_{59}NO_{15}$	7.3	$[M+H]^+$	756.50591	
**OTa**	$C_{11}H_9ClO_5$	7.74	$[M+H]^+$	257.02113	239.0105
**AFB1**	$C_{17}H_{12}O_6$	7.93	$[M+H]^+$	313.07066	285.0757
**U-13C17-AFB1**	$^{13}C_{17}H_{12}O_6$	7.93	$[M+H]^+$	330.1277	
**HT-2**	$C_{22}H_{32}O_8$	8.1	$[M+H]^+$	425.21699	263.1279
**b-ZEL**	$C_{18}H_{24}O_5$	8.36	$[M+H]^+$	321.16965	285.1485
**a-ZEL**	$C_{18}H_{24}O_5$	8.77	$[M+H]^+$	321.16965	285.1485
**T-2**	$C_{24}H_{34}O_9$	9.07	$[M+Na]^+$	489.2095	245.1175
**OTA**	$C_{20}H_{18}ClNO_6$	9.36	$[M+H]^+$	404.0895	239.0106
**U-13C20-OTA**	$^{13}C_{20}H_{18}ClNO_6$	9.36	$[M+H]^+$	424.15664	
**ZEN**	$C_{18}H_{22}O_5$	9.45	$[M+H]^+$	319.154	187.0754
**DON**	$C_{15}H_{20}O_6$	4.14	$[M+H]^+$	297.13326	203.1065
**U-13C15-DON**	$^{13}C_{15}H_{20}O_6$	4.14	$[M+H]^+$	312.18359	
